# Time-Gated Optical Projection Tomography Allows Visualization of Adult Zebrafish Internal Structures

**DOI:** 10.1371/journal.pone.0050744

**Published:** 2012-11-19

**Authors:** Luca Fieramonti, Andrea Bassi, Efrem Alessandro Foglia, Anna Pistocchi, Cosimo D'Andrea, Gianluca Valentini, Rinaldo Cubeddu, Sandro De Silvestri, Giulio Cerullo, Franco Cotelli

**Affiliations:** 1 Department of Physics, Politecnico di Milano, Milano, Italy; 2 Department of Bioscience, Università degli Studi di Milano, Milano, Italy; 3 Istituto di Fotonica e Nanotecnologie, Consiglio Nazionale delle Ricerche, Milano, Italy; 4 Italian Institute of Technology (IIT), Milano, Italy; Institut Curie, France

## Abstract

Optical imaging through biological samples is compromised by tissue scattering and currently various approaches aim to overcome this limitation. In this paper we demonstrate that an all optical technique, based on non-linear upconversion of infrared ultrashort laser pulses and on multiple view acquisition, allows the reduction of scattering effects in tomographic imaging. This technique, namely *Time-Gated Optical Projection Tomography* (TGOPT), is used to reconstruct three dimensionally the internal structure of adult zebrafish without staining or clearing agents. This method extends the use of Optical Projection Tomography to optically diffusive samples yielding reconstructions with reduced artifacts, increased contrast and improved resolution with respect to those obtained with non-gated techniques. The paper shows that TGOPT is particularly suited for imaging the skeletal system and nervous structures of adult zebrafish.

## Introduction

During the last decade, three-dimensional optical imaging techniques have gained more and more importance in biological research, because they allow fast and effective visualisation of complex structures inside the sample volume, giving access to functional information too. The need to image whole embryos and specimens on the millimeter scale non-invasively has fostered the development of volume reconstruction methods like, among the others, *Selective Plane Illumination Microscopy* (SPIM) [1] and *Optical Projection Tomography* (OPT) [2], [3]. These techniques, with respect to confocal or two-photon microscopies, can image larger volumes in relatively short measurement times, enabling their application to in vivo imaging.

Recently, the zebrafish animal model (*Danio rerio*) has attracted the interest of developmental biology and cancer research, thanks to its unique features. As a matter of fact, the zebrafish provides ease of handling and drug administration, feasibility of forward and reverse genetics, high transparency at embryo stage and complexity of its circulatory system [4]. As a result, it is not surprising that zebrafish is becoming a model of choice, on the one hand for studying embryogenesis and organ development, on the other hand for understanding disease mechanisms and for testing drug efficacy. Consequently, there is a tremendous quest for novel imaging modalities that allow the visualization of zebrafish internal structures *in vivo*. Common practice is to chemically clear the specimen *ex vivo* with toxic chemicals and to compare the results coming from samples at different development stages. However, this approach is time consuming and requires a large number of specimens. Instead, time lapse measurements can be performed *in vivo* on the same sample, ensuring that physiological conditions are respected. In this case, the light scattering exhibited by uncleared tissues can pose severe limitations on imaging depth, making 3D image reconstruction unfeasible for large specimens, like adult zebrafish.

The issue of reducing light scattering during tomographic imaging has been addressed by different imaging modalities, in particular by photoacoustic, phase-control and time-gated imaging. Photoacoustic microscopy is a technique that combines the optical absorption contrast with the high resolution that comes from ultrasonic wave detection to reconstruct internal features of specimens. However, many deep embedded structures do not absorb enough light to generate a detectable photoacoustic signal in physiological conditions [5]. Phase-control imaging is based on spatial wavefront shaping of the illuminating light. Thanks to interference phenomena, it is possible to control the wavefront transmitted through a strongly scattering sample. However, this technique is at a preliminary stage and it has been proved only on very small phantoms [6]. Finally, time-gated imaging is a technique able to select a limited temporal portion of a light pulse that has passed through a diffusive sample. This method allows the detection of quasi-ballistic photons, thus rejecting multiply scattered ones, which are responsible for blurring and resolution reduction in the transmission image.

The idea of placing a time window during signal detection for improving spatial resolution was suggested in the 1990's, fostered by the study of Yoo and Alfano (1990) about ultrafast light pulse propagation in random media [7]. Since then, a number of different gating modalities have been demonstrated. However, only recently has it been applied to tomographic reconstruction of biological specimens. A high-speed gated image intensified CCD is usually employed to obtain gating windows with a temporal duration of hundreds of picoseconds, which is limited by the electronic response of the camera. As a result, the resolution reached with these systems is around 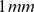 and, therefore, only diffusing specimens in the centimeter scale can be investigated [8], [9].

We recently proposed a technique, referred to as Time-Gated Optical Projection Tomography (TGOPT), which can be classified as a non-linear optical-gating technique, like those based on the Kerr effect or parametric amplification [10], [11]. TGOPT uses non-linear upconversion for gating the optical signal in the femtosecond time scale [12].

Here we report on the application of TGOPT to image an adult zebrafish (*casper* mutant), which does not present skin pigmentation [13]. We demonstrate the capability of the technique to tomographically reconstruct the internal structures of the specimen, especially skeletal and nervous systems. This result has been achieved in a non-invasive way, which is usually a challenge for optical tomography, because of the strong light scattering of the sample.

## Results and Discussion

**Figure 1:**
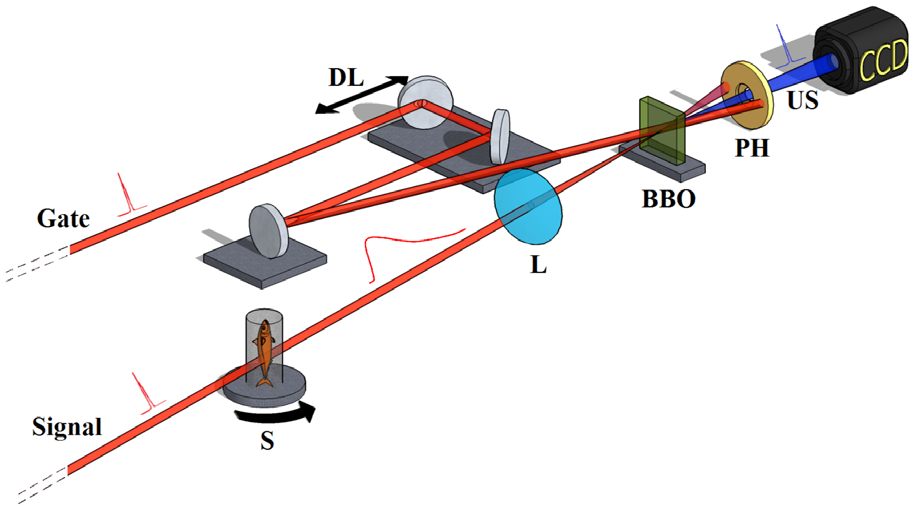
Physical operating principle of TGOPT. The *gate* pulse, which is a replica of the *signal* one, is properly synchronized thanks to a delay-line (DL). The *signal* is sent through the sample (S) and broadens its temporal length due to scattering. It is then focused with a lens (L) on the BBO crystal. Finally, the generated upconverted signal (US) is selected with a pin-hole (PH) and captured by a CCD camera.

The time-gated mechanism at the base of TGOPT is schematically shown in Fig. 1. An ultrashort laser pulse, called “signal”, is sent through the biological sample. Tissue scattering properties broaden its temporal duration from the femtosecond to the picosecond time scale [14]. The transmitted light is then focused with a lens on a non-linear optical crystal. The “gate” pulse, which is a replica of the “signal”, is sent to the non-linear crystal through a delay line, which allows synchronization of the two pulses. Consequently, a sum frequency beam is generated by the crystal only when they overlap temporally. By synchronizing the “gate” pulse with the leading edge of the “signal”, primarily ballistic photons are selected and upconverted for CCD detection through a bandpass filter. For more details on the TGOPT setup, refer to *Materials and Methods* and *Supporting Information* Text S1.

Concerning tomographic reconstruction, TGOPT makes use of the same principle as OPT, which consists in a mathematical processing of optical parallel projections recorded at different view angles (see *Materials and Methods*). It is worth noting that the TGOPT setup can be easily converted into an OPT system with just minor modifications (see *Supporting Information* Text S1), thus ensuring comparability between images obtained with the two modalities.

The contrast in TGOPT is given by the optical attenuation, which stems from both absorption and scattering properties of the tissues. The absorbing structures decrease the light intensity at the detector and, as in conventional OPT, appear in the projections as dark regions. On the other hand, optical scattering changes only the direction of the propagating photons and, in OPT, this gives rise to a diffused bright background. Conversely, in TGOPT these photons are rejected and the structures that present high scattering are detected again as dark regions in the projections.

**Figure 2:**
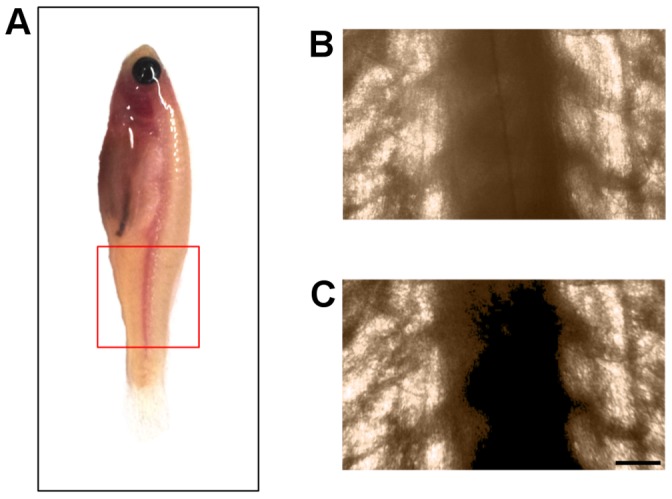
Resolution and contrast improvement with TGOPT. (a) Photo of the adult 22 mm long zebrafish used for the measurements. The red box shows the region considered for analyses. (b) Parallel projection captured by OPT. (c) Parallel projection obtained with an *early*-TGOPT. The early gate rejects multiple scattered photons, improving resolution and contrast. The effect is evident for the vertebral column in the center of the images, which should be displayed as a black structure if only ballistic photons were present, because of its large attenuation properties. Scale bar for (b)-(c) is 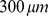.

We performed TGOPT analysis on a 3 months-post-fertilization adult male zebrafish, 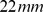 long. The laser fluence used in our experiment was well below the ablation threshold of tissues (see *Materials and Methods*) and, due to the low laser repetition rate (1 kHz), heat accumulation effects and thermal damage were negligible. The region considered for tomographic reconstruction is the trunk, between the swimbladder and the tail (Fig. 2(a), red box), where sample dimensions are 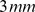 in width and 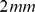 in thickness. In order to ease the rotation of the specimen during tomography, we used a protocol for its inclusion in agarose gel (see *Materials and Methods*).

Figures 2(b)–(c) present parallel projections of the sample, as captured by the CCD, for both OPT and TGOPT. Figure 2(c) shows the projection measured with TGOPT when the 100 fs temporal gating window is placed on the leading edge of the signal laser pulse, just before the peak. We will refer to it as an *early*-TGOPT. It can be compared to the projection obtained with OPT, depicted in Fig. 2(b). Although the TGOPT technique displays its full power in the 3D reconstructions, it is possible to notice an overall improvement of the image contrast. The effect is particularly evident for the vertebral column, which is in the center of the images. Since it is a strongly attenuating structure, it should be displayed as a completely black feature in projective images. However, photons that come from the spine sides get scattered many times by the nearby tissue during their travel. If no gating method is applied, they are detected by the camera as if they had passed straight through the spine, creating inconsistencies during tomographic reconstruction. This is the case of OPT, where some blurring is superimposed to the spine. Instead, TGOPT removes the majority of scattered photons, thus displaying the vertebral column with much higher contrast.

As already said, the purpose of TGOPT is to place the gating window on the first part of the “signal”. However, the synchronization delay of the “gate” can be precisely controlled and it can be changed in order to find the one which maximizes the contrast. This makes the technique very flexible. As a matter of fact, one has to face a trade-off between the selection of ballistic photons and the need to receive a sufficient signal intensity on the CCD for an acceptable signal-to-noise ratio. More details on this topic can be found in *Supporting Information* Text S1 and Fig. S3.

**Figure 3:**
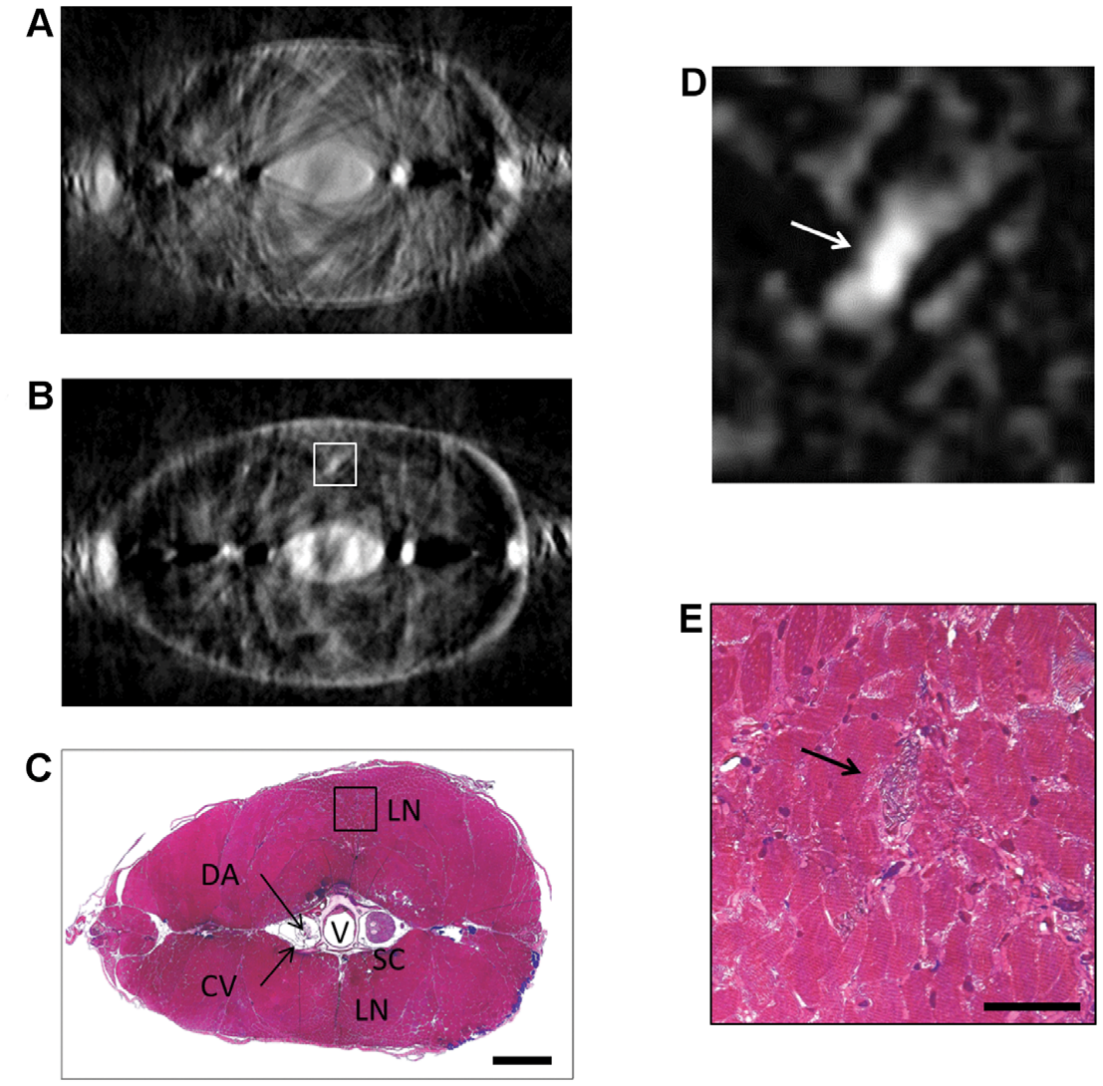
Comparison between transverse sections. (a) Virtual transverse section reconstructed by OPT. (b) Virtual transverse section reconstructed by *early*-TGOPT. (c) Correspondent histological section for comparison. V: vertebra, SC: spinal cord, LN: lateral nerve, DA: dorsal aorta, CV: cardinal vein. (d) Closeup of the highlighted area in the *early*-TGOPT section. (e) Closeup of the highlighted area in the histological section. The arrows point to the lateral nerve. Scale bar for (a)-(c) is 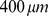; scale bar for (d)-(e) is 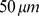.

When tomographic reconstruction is performed with TGOPT (see *Materials and Methods*), the contrast increase in single projections reduces the ill-posed nature of the inverse problem, thus giving an even more evident improvement in the resolution of the reconstructed volume with respect to OPT. Figure 3 presents transverse sections obtained with different techniques. The virtual section reconstructed by *early*-TGOPT is shown in Fig. 3(b). By comparing it to Fig. 3(c), which is the corresponding histological section of the specimen (see *Materials and Methods*), it is clear that all principal features are correctly revealed by TGOPT. The vertebral column, which is the innermost structure of the sample, can be precisely identified and the ring-like shape of the sectioned vertebra is clearly visible. The dorsal aorta and the cardinal vein can be located as well, even if they are very close to the spine. The spinal cord can be well detected too, with a contrast similar to that of the bones, because nervous structures show much more scattering than muscular tissue, due to their high lipidic content.

By comparing the OPT reconstructed transverse slice in Fig. 3(a) with the *early*-TGOPT one (Fig. 3(b)), it is plain to see the drastic artifact reduction that occurs when the contribute of multiply scattered photons is removed. It must be noted that the blurring effect, due to inconsistencies in the data, prevents OPT from the precise localization of internal structures. Nevertheless, a few artifacts still remain in TGOPT: some stripes can be noted around the skin of the specimen and black spots are present inside the volume. The latter effect is similar to the “metal artifact”, present in x-ray computed tomography [15]. Due to the limited dynamic range of the detector, the structures inside the volume exhibiting strong light attenuation (e.g. the bones of the zebrafish) break the linearity assumption at the basis of the tomographic reconstruction. This phenomenon generates inconsistencies in the data, which in turn create black regions around these structures when filtered back-projection is applied.

Remarkably, TGOPT is able to detect two lateral white spots, which correspond to the posterior lateral line nerves of the zebrafish. They disappear almost completely in the OPT reconstruction, due to the presence of a large number of artifacts. These nerves innervate the lateral lines, which are sensory organs typical of fish and amphibians. Lateral lines can react to water motion and are involved in prey detection, predator avoidance and in schooling behaviour [16].

Nervous tissue and bones are the most contrasted structures in TGOPT because of their light attenuation properties. If an object inside the volume attenuates light in the same way as the surrounding tissue, it is impossible to distinguish it, no matter its size. The resolution of TGOPT depends on several factors which include the imaging lens resolving power (approximately 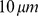 for the 1X objective used), aberrations caused by the non-linear conversion, small movements of the specimen during acquisition, the scattering properties of the sample. We estimate that the spatial resolution is better than 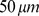, because this is the size of lateral nerves, which are the smallest well-contrasted details of the image. Figure 3(d) and Fig. 3(e) show a close up of these structures for *early*-TGOPT and histological sections, respectively.

**Figure 4:**
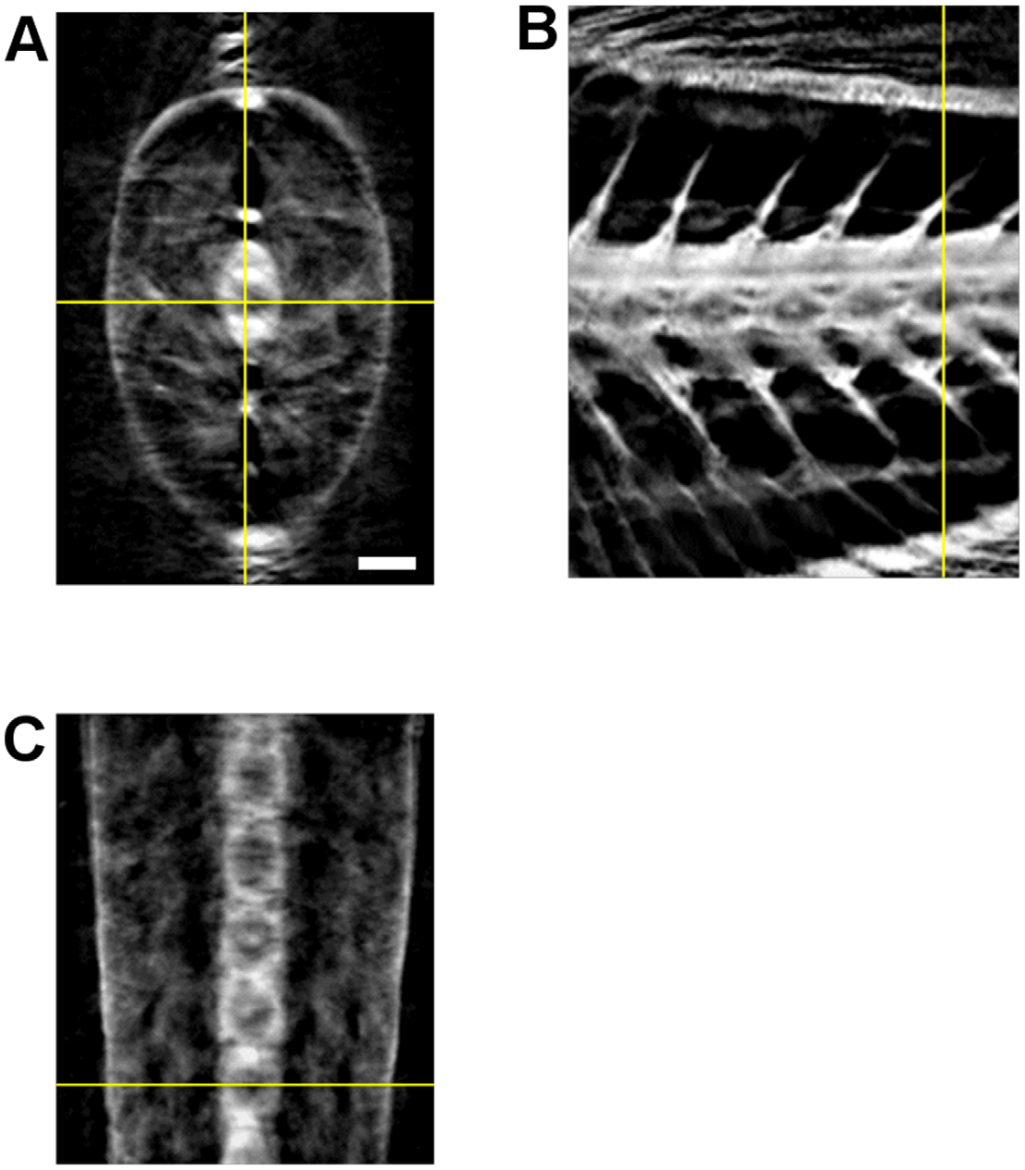
Orthogonal views of the TGOPT reconstructed volume. (a) Transverse, (b) sagittal, and (c) coronal sections. Scale bar is 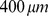.

Once the three-dimensional volume has been reconstructed, it can be sectioned along orthogonal planes, e.g. transverse, sagittal and coronal ones. This can be useful to visualize and analyze different details of the sample that do not lie completely in a single horizontal slice, like the vertebral column. The zebrafish model is currently used to study bone development, spine malformations and degenerative osseous diseases [17], [18]. In this case, tomographic reconstruction of adult zebrafish could be performed with Micro-CT with approximately 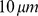 resolution [19]. However, since this technique uses x-rays to reconstruct density variations inside the tissue, it cannot give spectroscopic information and it cannot be used for *in vivo* applications, because a proper staining or metal-containing agents are required for gaining a good contrast [20]. Figure 4 shows the ability of TGOPT to allow analysis of the vertebral column as a whole. The planes used to build the orthogonal projections are chosen to pass exactly through the center of the vertebrae. In the sagittal view (Fig. 4(b)) it is possible to see how the spine is placed with respect to the spine chord and the main blood vessels. Instead, the coronal view (Fig. 4(c)) shows how the vertebrae are arranged in the fish trunk, which can be useful if one wants to detect possible malformations or injuries of the skeletal structure.

### Conclusions

In this paper, we have demonstrated that TGOPT enables tomographic reconstruction of adult zebrafish internal structures in the trunk region by reducing drastically the effect of scattering. As a matter of fact, tomographic reconstruction of adult zebrafish has already been demonstrated with photoacoustic tomography, but, because of the thickness of the sample, labelling was necessary in order to enhance the optical absorption of deep structures, e.g. the vertebral column, thus increasing the photoacoustic signal emitted to a detectable level [5]. Instead, TGOPT is able to visualize different features of the sample without staining methods or clearing agents, thanks to a non-linear time-gating process. We have also compared this technique with OPT, whose contrast and resolution are greatly spoiled by the strong optical scattering that characterizes thick biological samples. The study shows not only that the overall contrast is increased, but also that artifacts are reduced and some previously unobservable structures, like the lateral nerves, become visible. From these results, we believe that TGOPT can be successfully employed for tomographic *in vivo* measurement and analysis of the skeletal and nervous systems of zebrafish.

The power of TGOPT is that it is a non-invasive all-optical technique that can work with different wavelengths in the near infrared region of the electromagnetic spectrum. As a result, our future work will be dedicated to implement a multispectral version of it. On one side, this approach can result in artifact reduction and in an overall improvement of the tomographic reconstruction, thanks to the redundancy of data taken at different wavelengths; on the other side, it should help in increasing the contrast, through the analysis of the slight variations in the absorption coefficient as a function of the wavelength for all the structures. Even if time gated methods cannot be used to reveal fluorescence signals due to the long fluorescence lifetimes, it is likely that the reconstruction provided by TGOPT could be used to predict the scattering effect on fluorescence images with an appropriate mathematical model. Future work will be dedicated to the combination of the technique with fluorescence imaging.

## Materials and Methods

### 0.1 Ethical Statement

All the experimental protocols were reviewed and approved by the Institutional Review Board at the Department of Biosciences of University of Milan (Pr. 012-01). They have been conducted in accordance with national (Italian D.lgs. 116/92), European (European Directive 86/609/EEC on the protection of animals used for scientific and other experimental purposes) and international laws and regulations controlling experiments on live animals. All measurements were performed under tricaine anesthesia and all efforts were made to minimize animal suffering and the number of animals used.

### 0.2 Zebrafish Handling Protocols

All experiments were performed on 3 months-old *casper* mutant adult zebrafish 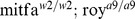 housed in the zebrafish facility of Università degli Studi di Milano, Dipartimento di Bioscienze and maintained under standard conditions of temperature 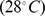 and photoperiod (14-hour light/10-hour dark). Ten fish of the same sex were kept in tanks containing 1 L of water.

### 0.3 Sample Preparation

To perform measurements, the specimen was anaesthetized with tricaine (MS-222, 160 mg/l) and included in a 2% low melting point agarose cylinder (1 cm diameter), as suggested elsewhere [5]. Particular attention has been put on centering the fish inside the cylinder. After inclusion, the agarose cylinder was glued at one end to a metallic support, which had been fixed to the rotating stage that allows tomography. The agarose cylinder was placed in a cubic cuvette, with a side size of 30 mm, filled with water. The face of the cuvette exposed to light was oriented orthogonal to the laser beam.

### 0.4 Histological Sections

Fish were sacrificed with an overdose of tricaine (MS-222, 960 mg/l), as suggested by AVMA (American Veterinary Medical Association) and approved by European and Italian legislation. Sacrificed fish were fixed overnight at 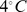 with 1.5% glutaraldehyde and 4% paraformaldehyde in 0.1 M sodium cacodylate buffer pH 7.3. They were rinsed in the same buffer and post-fixed for 1 hour in sodium-cacodylate-buffered 1% osmium tetroxide. The samples were then dehydrated in a graded ethanol series, transitioned to propylene oxide and embedded in Epon 812-Araldite. Sections with a thickness of 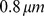 were obtained using a Reichert Ultracut E instrument. The sections were stained with gentian violet and basic fuchsin and photographed with Leica DM 6000B microscope, equipped with a Leica DFC320 digital camera.

### 0.5 TGOPT Operating Principle

The selection of quasi-ballistic photons is achieved by illuminating the specimen with ultrashort light pulses at a repetition rate of 1 kHz. The 100 fs pulses are produced by an amplified Ti:Sapphire, with a central wavelength of 800 nm and a diameter of 6 mm. Every pulse is split into two replicas, namely “signal” and “gate” pulses, and attenuated in intensity. The “signal” pulse is sent to the specimen with an energy of 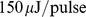, corresponding to a fluence on the target of 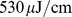 ^2^. This value is orders of magnitude lower than the ablation threshold for tissues, which is about 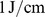 ^2^, depending on the specific tissue [21], [22]. During propagation within a few millimeters of biological tissue, the pulse temporal duration is broadened to tens of picoseconds because of the scattering [14]. The “gate” pulse, instead, is delayed for synchronization purposes and then recombined with the “signal” on a 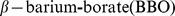 crystal. As a consequence, a sum frequency pulse is generated from the BBO, provided that the two pulses overlap both spatially and temporally. The “gate” pulse selects only the first part of the “signal”, thus letting quasi-ballistic and single scattered photons within the 100 fs temporal window to be upconverted and to be captured by the CCD. For a detailed description of the imaging setup, see *Supporting Information* Text S1 and Fig. S1, while examples of projective images captured with and without the temporal gating can be seen in Fig. S2.

### 0.6 Tomographic Reconstruction and Image Processing

In order to perform tomographic reconstruction, 400 projections of the sample uniformly distributed over 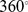 were captured. This process took less than 3 minutes. We used a backprojection algorithm based on the inverse Radon transform. This is standard for Computed Tomography and OPT, and it is well suited for TGOPT, because it assumes that light propagates through the sample along straight lines. This corresponds to the behaviour of ballistic photons. We have written the whole reconstruction algorithm in Matlab (MathWorks). The visualization of the reconstructed data was done with the public domain software ImageJ (NIH). Both OPT and TGOPT reconstructions underwent the same identical histogram normalization and background reduction to preserve comparability between them.

## Supporting Information

Figure S1 **Sketch of the TGOPT imaging setup.** BS: beamsplitter; VA: variable attenuator; D: rotating diffuser; OBJ: objective lens; PCS: polarizing cube splitter; GTP: Glan-Taylor prism; IF: interference filter.
(TIF)

Figure S2 **Parallel projections of the sample for the evaluation of the half-wave plate effect.** (a) and (b) are respectively OPT and TGOPT projections of the specimen in the presence of the half-wave plate. (c) and (d) are projections obtained without the half-wave plate for OPT and TGOPT, respectively.
(TIF)

Figure S3
**Synchronization of the temporal window over the “signal” pulse. (**a) Three representative positions of the gating window are depicted with respect to the indicative transmitted signal curve. (b) Parallel projection corresponding to gate T1. Only ballistic photons are captured, but the image has a very low contrast due to poor SNR. (c) Parallel projection corresponding to gate T2, delayed by 660 fs with respect to T1. Higher SNR is achieved, while multiply scattered photons are still rejected. (c) Parallel projection corresponding to gate T3, delayed by 1160 fs with respect to T1. Low SNR and strongly scattered photons spoil completely the image. Scale bar for (b)-(d) is 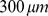.(TIF)

Text S1 Supporting Information main text.
(DOC)
