# Supplementary figures and images for: Time-Gated Optical Projection Tomography Allows Visualization of Adult Zebrafish Internal Structures
Source: PLoS One. 2012 Nov 19;7(11):e50744. doi: 10.1371/journal.pone.0050744 (PMC3501464; doi:10.1371/journal.pone.0050744)

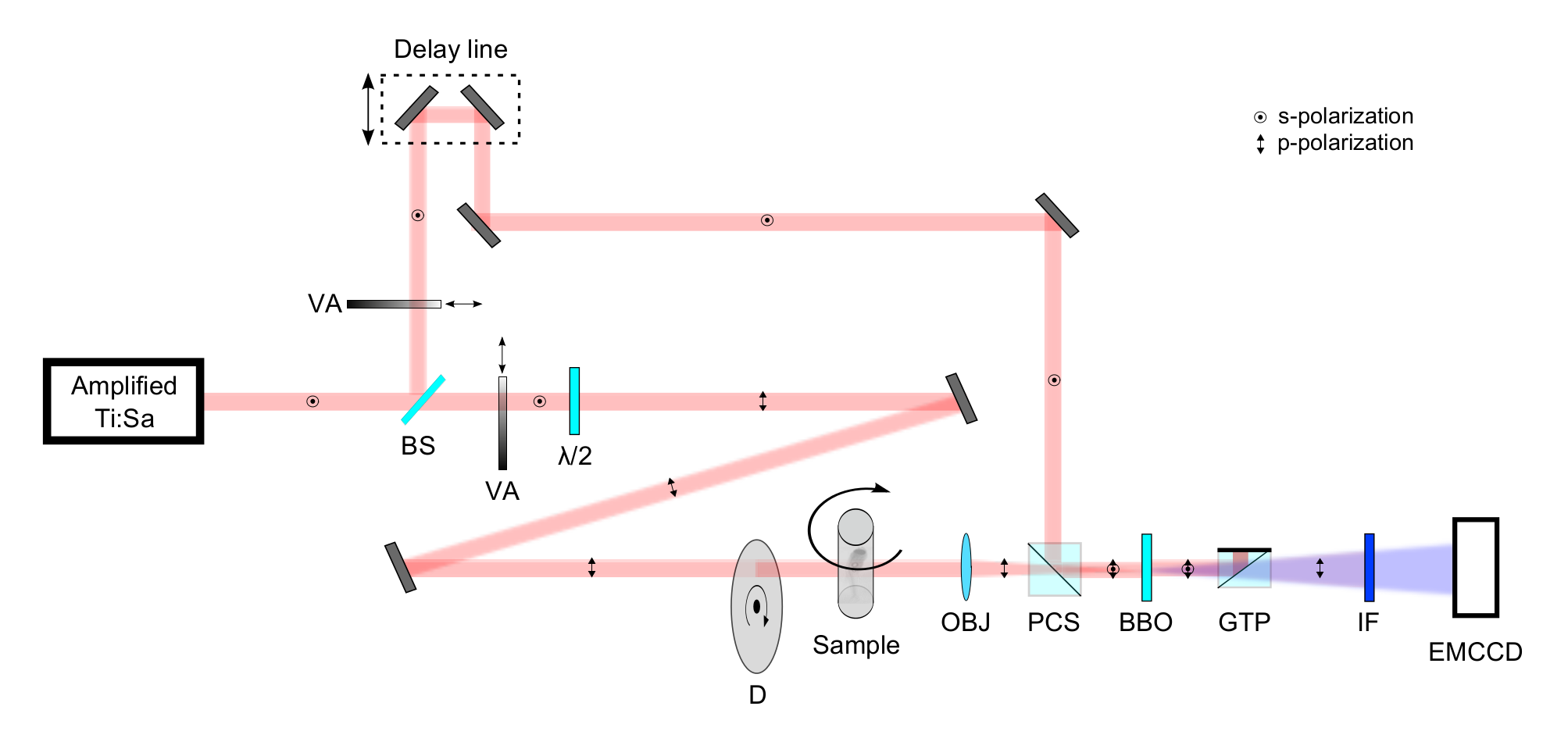

Supplement: Figure S1 — Sketch of the TGOPT imaging setup. BS: beamsplitter; VA: variable attenuator; D: rotating diffuser; OBJ: objective lens; PCS: polarizing cube splitter; GTP: Glan-Taylor prism; IF: interference filter. (TIF) [file pone.0050744.s001.tif]

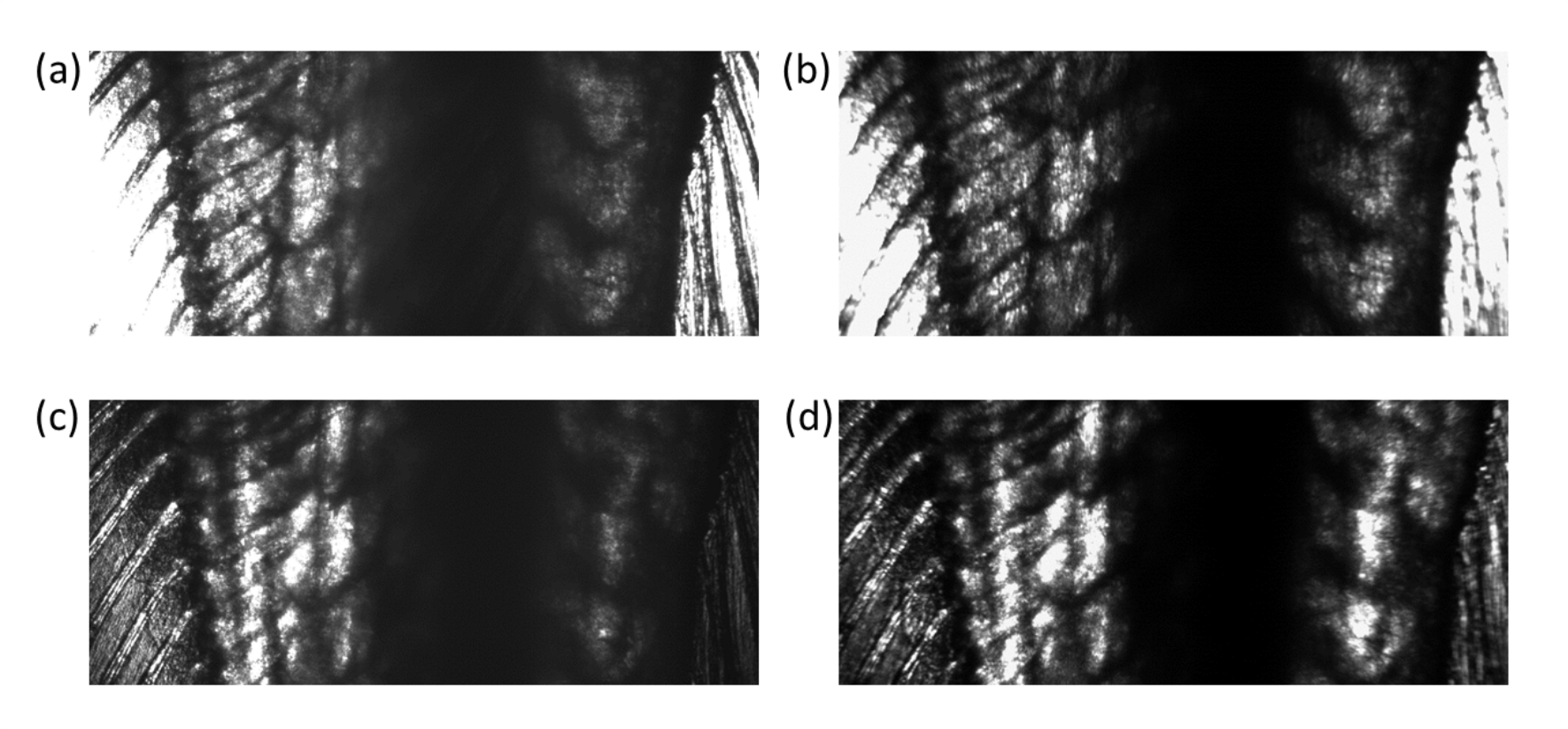

Supplement: Figure S2 — Parallel projections of the sample for the evaluation of the half-wave plate effect. (a) and (b) are respectively OPT and TGOPT projections of the specimen in the presence of the half-wave plate. (c) and (d) are projections obtained without the half-wave plate for OPT and TGOPT, respectively. (TIF) [file pone.0050744.s002.tif]

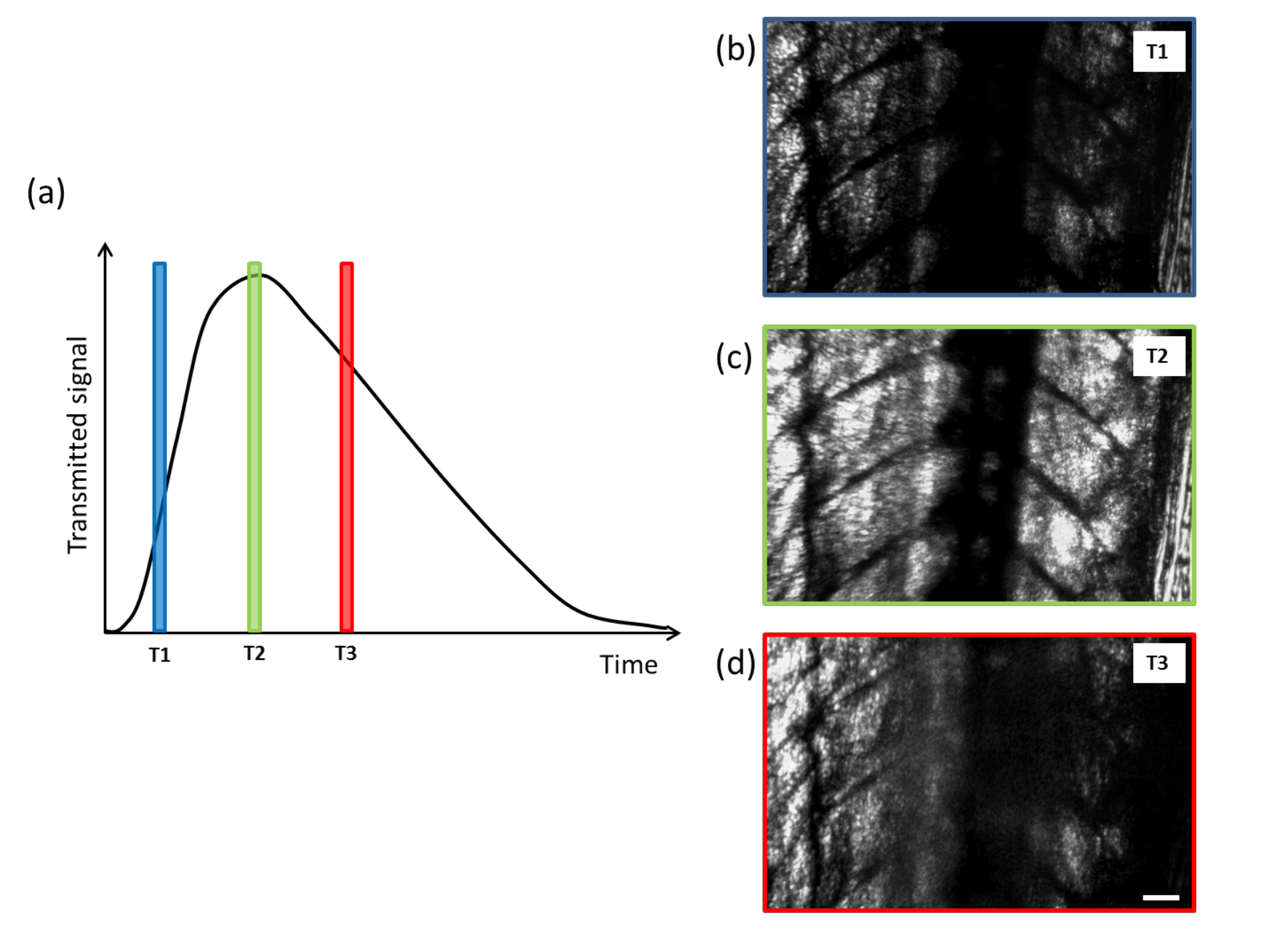

Supplement: Figure S3 — Synchronization of the temporal window over the “signal” pulse. (a) Three representative positions of the gating window are depicted with respect to the indicative transmitted signal curve. (b) Parallel projection corresponding to gate T1. Only ballistic photons are captured, but the image has a very low contrast due to poor SNR. (c) Parallel projection corresponding to gate T2, delayed by 660 fs with respect to T1. Higher SNR is achieved, while multiply scattered photons are still rejected. (c) Parallel projection corresponding to gate T3, delayed by 1160 fs with respect to T1. Low SNR and strongly scattered photons spoil completely the image. Scale bar for (b)-(d) is . (TIF) [file pone.0050744.s003.tif]
