# Supplementary material for: Time-Gated Optical Projection Tomography Allows Visualization of Adult Zebrafish Internal Structures
Source: PLoS One. 2012 Nov 19;7(11):e50744. doi: 10.1371/journal.pone.0050744 (PMC3501464; doi:10.1371/journal.pone.0050744)
Supplement: Text S1 — Supporting Information main text. (DOC) [file pone.0050744.s004.doc]

**SUPPORTING INFORMATION**

**Imaging Setup**

The TGOPT imaging setup used for the experiment is an improved version of the one we described in a previous paper [1]. It is depicted schematically in Fig. S1. The light source is an amplified Ti:Sapphire laser, which emits s-polarized pulses with a temporal duration of about 100 fs, centered at 800 nm, with an energy per pulse of about 600 µJ at a repetition rate of 1 kHz. After collimation to a 6 mm beam waist, the pulse is divided by a beam splitter into a *“signal”* pulse and a *“gate”* pulse. Both are reduced to the desired energy by variable attenuators. The polarization of the *“signal”*  beam is rotated by 90° into a p-polarization by a half-wave plate. After passing through a rotating diffuser for homogenizing the beam profile and reducing interference effects, which spoil the final image quality, the *“signal”*  pulse hits the sample and then it is focused on a BBO crystal (Type II, 1 mm thick) with the use of a 1x microscope objective. Instead, the *“gate”* pulse goes to a motorized delay line that allows synchronization of the two pulse replicas. Thanks to a polarizing cube splitter, it is eventually recombined to the *“signal”*  pulse on the BBO crystal. Here, sum frequency generation takes place, producing a p-polarized image of the sample at 400 nm. Before reaching the CCD camera, the *“gate”* pulse is rejected with a Glan-Taylor prism and the *“signal”*  beam at 800 nm is eliminated with interference filters. For all the acquired images, the integration time on the CCD was set to 100 ms.

While doing the measurement, we preferred to remove the half-wave plate from the signal path to prevent image saturation on the sides of the fish, which spoilt the image irremediably due to cross-talk effects between pixels of the camera. As a result, only those photons, whose polarization was rotated by the diffuser and the sample, generate the second-harmonic image. This solution removes completely the problem of saturation. As can be seen in Fig. S2, the use or non-use of the half-wave plate changes only slightly the contrast of some structures, which becomes somewhat similar to the one displayed in birefringence microscopy [2]. Nevertheless, the improvement on the image by TGOPT is exactly the same and justifies the application of the technique in both cases. In the future different solutions to the saturation problem based on a high-dynamic range measurement approach [3] could be investigated.

Lastly, in order to perform normal OPT measures with the TGOPT system, it is only necessary to turn off the temporal gating by physically blocking the gating pulse and to remove the interference filters, thus letting all the transmitted light to be integrated on the CCD. Note that the camera position must be adjusted, because the image planes are different for the two wavelengths considered.

**Tuning of the temporal delay**

Thanks to the motorized delay line, the temporal position of the gating window over the “*signal”* pulse can be precisely adjusted, as schematically depicted in Fig. S3(a). This is necessary to obtain the best parallel projections, taking into account the trade-off between the selection of ballistic photons and the intensity of the transmitted light. In theory, one should place the gating window on the very beginning of the *“signal”* pulse to collect only those photons that do not undergo scattering events, as shown in Fig. S3(b). However, since the laser intensity must be well below the threshold for tissue damage, the actual number of these photons is very low and does not give a sufficient signal-to-noise ratio (SNR) after upconversion. As a result, it is more convenient to place the gating window just before the peak of the transmitted light, because it gives a higher SNR and it allows the rejection of the multiply scattered photons, which arrive after the peak. In this case, the CCD captures mainly quasi-ballistic photons, also known as *snake* photons. This is the strategy we followed for TGOPT measurements. The resulting projection is presented in Fig. S3(c). Instead, Fig. S3(d) shows the effect of choosing a late gate, i.e. placing the gating window over the trailing edge of the *“signal”* pulse. Here, not only the SNR is low, but the blurring effect due to multiply scattered photons is evident.

**REFERENCES**

**[1]** A. Bassi, D. Brida, C. D'Andrea, G. Valentini, R. Cubeddu, et al., “Time-gated optical projection tomography”, Opt Lett 35: 2732-2734 (2010).

**[2]** J. R. Guyon, J. Goswami, S. J. Jun, M. Thorne, M. Howell, T. Pusack, G. Kawahara, L. S. Steffen, M. Galdzicki, and L. M. Kunkel, “Genetic isolation and characterization of a splicing mutant of zebrafish dystrophin”, Hum. Mol. Genet. 18(1): 202-211 (2009).

**[3]** P. Fei, Z. Yu, X. Wang, P. Lu, Y. Fu, Z. He, J. Xiong, and Y. Huang, "High dynamic range optical projection tomography (HDR-OPT)", Opt. Express 20, 8824-8836 (2012).
